# Ultra-High Field MRI Post Mortem Structural Connectivity of the Human Subthalamic Nucleus, Substantia Nigra, and Globus Pallidus

**DOI:** 10.3389/fnana.2016.00066

**Published:** 2016-06-16

**Authors:** Birgit R. Plantinga, Alard Roebroeck, Valentin G. Kemper, Kâmil Uludağ, Maartje Melse, Jürgen Mai, Mark L. Kuijf, Andreas Herrler, Ali Jahanshahi, Bart M. ter Haar Romeny, Yasin Temel

**Affiliations:** ^1^Department of Biomedical Image Analysis, Eindhoven University of TechnologyEindhoven, Netherlands; ^2^Department of Translational Neuroscience, Maastricht UniversityMaastricht, Netherlands; ^3^Department of Cognitive Neuroscience, Maastricht UniversityMaastricht, Netherlands; ^4^Department of Neuroanatomy, Heinrich-Heine-University DüsseldorfDüsseldorf, Germany; ^5^Department of Neurology, Maastricht University Medical CenterMaastricht, Netherlands; ^6^Department of Anatomy and Embryology, Maastricht UniversityMaastricht, Netherlands; ^7^Department of Neurosurgery, Maastricht University Medical CenterMaastricht, Netherlands

**Keywords:** subthalamic nucleus, substantia nigra, globus pallidus, post mortem, ultra-high field MRI, tractography

## Abstract

**Introduction:** The subthalamic nucleus, substantia nigra, and globus pallidus, three nuclei of the human basal ganglia, play an important role in motor, associative, and limbic processing. The network of the basal ganglia is generally characterized by a direct, indirect, and hyperdirect pathway. This study aims to investigate the mesoscopic nature of these connections between the subthalamic nucleus, substantia nigra, and globus pallidus and their surrounding structures.

**Methods:** A human post mortem brain specimen including the substantia nigra, subthalamic nucleus, and globus pallidus was scanned on a 7 T MRI scanner. High resolution diffusion weighted images were used to reconstruct the fibers intersecting the substantia nigra, subthalamic nucleus, and globus pallidus. The course and density of these tracks was analyzed.

**Results:** Most of the commonly established projections of the subthalamic nucleus, substantia nigra, and globus pallidus were successfully reconstructed. However, some of the reconstructed fiber tracks such as the connections of the substantia nigra pars compacta to the other included nuclei and the connections with the anterior commissure have not been shown previously. In addition, the quantitative tractography approach showed a typical degree of connectivity previously not documented. An example is the relatively larger projections of the subthalamic nucleus to the substantia nigra pars reticulata when compared to the projections to the globus pallidus internus.

**Discussion:** This study shows that ultra-high field post mortem tractography allows for detailed 3D reconstruction of the projections of deep brain structures in humans. Although the results should be interpreted carefully, the newly identified connections contribute to our understanding of the basal ganglia.

## Introduction

The basal ganglia are a network of nuclei deep in the brain, consisting of the substantia nigra (SN), subthalamic nucleus (STN), globus pallidus (GP), and striatum as their core structures. The network plays a fundamental role in a wide range of processes related to motor, associative, and limbic functions (Middleton and Strick, 2000; Temel et al., 2005). Its key role is illustrated by diseases of the basal ganglia, such as Parkinson's disease (PD), where disruptions in the circuit lead to the typical motor, cognitive, and mood changes (DeLong and Wichmann, 2007; Obeso et al., 2014). Modulation by electrical stimulation of motor and limbic parts of the basal ganglia nuclei, such as the STN and GP, has substantial therapeutic effects in neuropsychiatric disorders, including PD, obsessive-compulsive disorder, and Tourette syndrome (Mallet et al., 2008; Schuepbach et al., 2013; Kefalopoulou et al., 2015).

In humans, the striatum can be divided into the caudate nucleus, putamen, and nucleus accumbens; the substantia nigra can be divided into a pars compacta (SNc) and a pars reticulata (SNr); and the globus pallidus into an internal (GPi) and an external part (GPe) and the ventral pallidum. Classically, the basal ganglia circuit consists of three pathways: the direct, the indirect, and the hyperdirect pathway (Nambu, 2011). In the direct pathway, the cortex projects onto the striatum, which directly connects to the GPi and SNr; known as the output structures of the basal ganglia. In the indirect pathway, the striatum also projects onto the GPi and SNr, but via a detour through the GPe and STN. Finally, in the hyperdirect pathway, the STN directly receives its input from the cortex and then transfers it to the GPi and SNr again. This model is largely based on histology, electrophysiology, and tracing studies in rodents (Gerfen, 1985; Parent and Hazrati, 1993) and monkeys (Nauta and Mehler, 1966; Carpenter and Peter, 1972; Alexander et al., 1986; Hoover and Strick, 1993; Yoshida et al., 1993; Wichmann et al., 1994; Haynes and Haber, 2013), and on anatomical and histological studies in dissected human brains (Mai et al., 1986; Cossette et al., 1999).

MRI techniques allow for imaging of the 3D anatomy, connectivity, and functionality of the intact human brain (Lehéricy et al., 2006; Di Martino et al., 2008; Brunenberg et al., 2012; Lenglet et al., 2012). However, although these studies provide human-specific contributions to the basal ganglia model, they can only infer the brain's connections at a coarse scale. Newly emerging ultra-high field (7 T) MRI scanners provide the opportunity to investigate the human brain at a higher resolution, higher contrast, and with less noise compared to the more widely available 1.5 and 3 T MRI scanners (Plantinga et al., 2014). Moreover, scanning of post mortem specimens allows for increasing the scan times, which leads to an additional improvement in image quality. In this study, we acquired ultra-high resolution structural and diffusion weighted images of a human post mortem brain specimen including the GP, STN, and SN at 7 T. Reconstructed pathways of these nuclei, demonstrate both the detailed wiring of a subset of the basal ganglia network in line with the established models, as well as novel details.

## Materials and methods

### Data acquisition

A unilateral formalin fixed human brain specimen was acquired from the Maastricht University Body donation program, Department of Anatomy and Embryology. The Local Medical Ethics Committee of Maastricht University has been informed about educational and research activities concerning donor material. A handwritten and signed codicil from the donor, drafted when still alive and well, is kept at the Department of Anatomy and Embryology, as is required by Dutch law for the use of cadavers for scientific research and education. The donor who was aged between 70 and 95 years, had no known neurological disorders. Time from death to formalin fixation was ~12 h. Three basal ganglia nuclei were completely enclosed in the post mortem piece: the STN, SN, and GP. Approximately 12 months after time of death, the specimen was embedded in phosphate buffered saline (PBS) for 7 days during which the PBS was refreshed 3 times, before being scanned in PBS on a 7 T MRI scanner (Magnetom 7 T, Siemens, Erlangen, Germany). The protocol consisted of a 3D gradient recalled echo (GRE) and a pulsed gradient spin echo segmented 3D diffusion weighted echo planar imaging (3D-dwEPI) sequence. The 3D-dwEPI was acquired with 3 lines-per-shot (segmentation factor 40), and a *b*-value of 2800 s/mm^2^. In addition to 8 b0-volumes, 60 diffusion directions were sampled. Given the scan time of 42 h, this allowed for a good identification of multiple fiber orientations while still being capable of scanning with a high spatial resolution of 0.5 × 0.5 × 0.5 mm^3^. Other scanning parameters are listed in Table 1.

**Table 1:** **Scan parameters**.

**Sequence**	**Resolution (mm^3^)**	**Matrix size**	**TE (ms)**	**TR (ms)**	**Flip angle (°)**	**Nr directions**	**Scan time (hh:mm:ss)**
GRE	0.3 × 0.3 × 0.3	192 × 192 × 208	11	37	8	n/a	00:13:04
3D-dwEPI	0.5 × 0.5 × 0.5	120 × 120 × 112	60	500	90	60	42:22:13

*GRE, gradient recalled echo; 3D-dwEPI, 3D diffusion weighted echo planar imaging; TE, echo time; TR, repitition time*.

### Data processing

The diffusion weighted images were corrected for eddy current-induced distortions and motion with FSL (Analysis Group, FMRIB, Oxford, UK; Jenkinson et al., 2012). The STN, SNc, SNr, GPi, and GPe were manually delineated from the GRE-image using ITK-SNAP software (Yushkevich et al., 2006). Because the striatum was only partially enclosed in the specimen, it was not considered in the analysis. These segmentations were then affinely coregistered to the corrected diffusion weighted space along with the GRE image using FSL's FLIRT (Andersson et al., 2007).

### Fiber tracking

Constrained spherical deconvolution-based probabilistic fiber tracking with a maximum harmonic order (lmax) of 8 was performed on the whole specimen with MRtrix (Tournier et al., 2012). Optimal parameter values were determined by visual inspection of the obtained fiber tracks in 3D; parameter sets that resulted in unrealistically short, long, straight, and/or noisy fiber tracks were excluded. This resulted in a step size of 0.05 mm, a minimum radius of curvature of 0.1 mm, a fiber orientation distribution (FOD) amplitude cutoff of 0.3, and an FOD amplitude cutoff for initiation of 0.01. Only fiber tracks intersecting the SN, STN, or GP were selected for further analysis, which resulted in a total of 416,832 fiber tracks.

### Connectivity analysis

To investigate the connectivity pattern of the included basal ganglia nuclei, various sets of fiber tracks were isolated, based on their direct or indirect connections to other manually segmented nuclei and known fiber bundles, including the red nucleus, ventral pallidum, anterior commissure, ansa lenticularis, internal capsule, sublenticular internal capsule, interthalamic adhesion, and the region of the medial lemniscus. The origin of these projections within the STN, SN, or GP was then mapped by computing for every voxel within these nuclei, the percentage of fibers running to each of its projection sites. Both these steps were performed with MRtrix (Tournier et al., 2012). Only projection sites with at least 1% of the fibers connecting to either of the basal ganglia nuclei were taken into account. In addition to these indirect connections, where a fiber track connects two structures via a detour through another structure, the direct connections between the SNc, SNr, STN, GPi, and GPe were also analyzed. To this end, custom written MATLAB (The MathWorks, Natick, U.S.A.) code was used to cut the original tracks into single track segments that directly connected the STN, SNc, SNr, GPi, or GPe with each other without passing through another of these five structures. To reduce the computation time of the analysis of the direct connections to less than 24 h, it was performed on only 9000 randomly selected original tracks, resulting in a total of 14,542 direct track segments.

### Description of the results

Anatomical orientations will be expressed in posterior-anterior, lateral-medial, and superior-inferior directions. Visualizations of the fiber bundles either have one color, or they follow the standard color coding for fiber orientation (posterior-anterior, green; lateral-medial, red; superior-inferior, blue).

## Results

**Figure 1:**
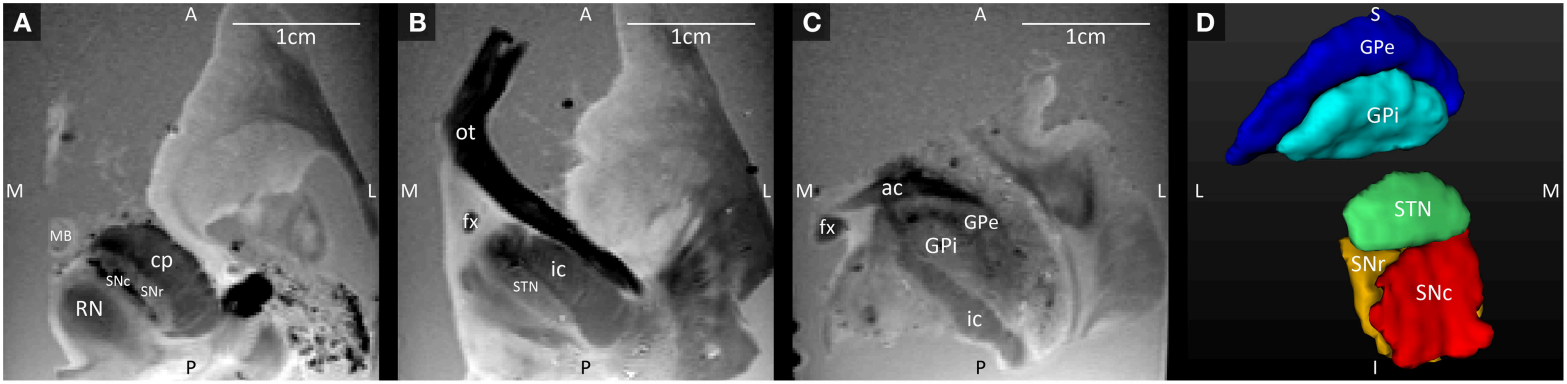
**(A–C)** Axial GRE images through the SNc and SNr, STN, and GPi and GPe, at three different levels ordered from the most inferior level **(A)** to the most superior level **(C)**. **(D)** 3D Visualization of the segmented structures. A, anterior; ac, anterior commissure; cp, cerebral peduncle; fx, fornix; GPe, external globus pallidus; GPi, internal globus pallidus; I, inferior; ic, internal capsule; L, lateral; MB, mammillary body; M, medial; ot, optic tract; P, posterior; RN, red nucleus; S, superior; SNc, substantia nigra pars compacta; SNr, substantia nigra pars reticulata; STN, subthalamic nucleus.

Three axial slices through the GRE images, with their anatomical annotations are shown in Figure 1. The hypointense SNc can be clearly separated from the SNr (Figure 1A), and the GPe and GPi are separated by the darker medial medullary lamina (Figure 1C). Figure 1D shows 3D representations of the segmented structures, which had the following volumes: STN = 100.5 mm^3^, SNc = 138.8 mm^3^, SNr = 142.6 mm^3^, GPe = 611.9 mm^3^, and GPi = 271.8 mm^3^.

### Connections

Table 2 gives an overview of structures directly and indirectly connected to the STN, SNc, SNr, GPe, and GPi. The number of tracks connecting two structures is expressed as a percentage of the total number of tracks through either the STN, SNc, SNr, GPe, or GPi. For example, 35.5% of all connections that course through the STN connect to the SN. The percentages of tracks through each structure add up to more than 100%, because a fiber track often connects one seed structure to multiple targets. Each track is counted once, even if it leaves a seed structure at two different borders. Connections to the GP are subdivided into connections going to the anteromedial and posterolateral halves of the GPe and GPi. A few of the reconstructed paths could not be reliably identified as known fiber bundles, and are therefore, in Table 2, described by their location.

**Table 2:** **Structures and fiber paths connected to the STN, SNc, SNr, GPe, and GPi**.

	**Seed structures**				
	**STN**	**SNc**	**SNr**	**GPe**	**GPi**
**Connecting structures**	**%**	**%**	**%**	**%**	**%**
Subthalamic nucleus		17.1	13.3	6.0	6.6
Substantia nigra	35.5			32.3	29.7
SN pars compacta	30.2		58.8	22.7	22.4
SN pars reticulata	28.6	71.4		26.7	20.2
Globus pallidus	29.9	51.4	43.7		
External GP	19.3	41.1	39.8		64.7
Anterior GPe	10.6	16.1	11.1		15.9
Posterior GPe	9.5	28.3	31.5		53.1
Internal GP	17.2	32.8	24.5	52.4	
Anterior GPi	17.0	30.3	15.9	30.3	
Posterior GPi	4.0	21.4	19.0	40.5	
Ventral pallidum				2.8	
Red nucleus				1.1	2.5
**Connecting fiber paths**	**%**	**%**	**%**	**%**	**%**
Anterior commissure				6.1	1.6
Ansa lenticularis	8.8	19.5	6.3	15.1	36.9
Inferior of SN	17.6	79.4	58.5	22.6	24.0
Internal capsule	94.7	89.7	98.2	78.5	73.1
Interthalamic adhesion					1.7
Lateral of IC		2.9	24.7		1.8
Medial to RN				1.8	1.7
Medial to STN	10.7			2.4	5.2
Sublenticular IC	10.5	9.3	12.0	21.7	29.6

*Each column shows the percentage of reconstructed tracks that connected the STN, SNc, SNr, GPe, or GPi (seed structures) with their targets. Only target structures connected with at least 1% of the seed's tracks were analyzed*.

### Direct connections within the basal ganglia

**Figure 2:**
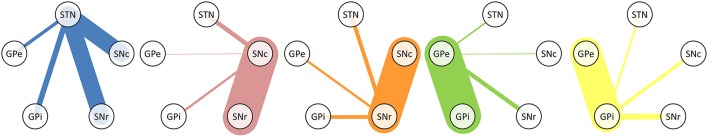
**Graphical representation of Table 3**. The line thickness is proportional to the relative number of fiber tracks connecting the structures.

The tracks described in Table 2 represent both direct and indirect connections. In indirect connections, tracks connect two structures via a detour through another structure. At least half of the original tracks connect more than two structures. Table 3 and Figure 2 focus solely on the direct connections between the STN, SNc, SNr, GPe, and GPi. The table is asymmetric because it shows the percentage of connections relative to the total number of direct connections of each structure. For example, of all direct connections between the STN and the four other involved structures 39.9% directly connected the STN with the SNc. Even though this step was performed on only a subset of 14,542 direct track segments, the number of direct connections between each pair of structures varied from 61 to 6683. It is therefore expected that performing this step on only a subset of the entire set of fiber tracks, will not have greatly influenced the results. The following sections (Section STN to Section GPe-GPi Connections), describe the anatomy of these connections (see also Supplementary Video 1).

**Table 3:** **Percentage of fibers directly connecting each structure**.

	**STN**	**SNc**	**SNr**	**GPe**	**GPi**
STN	X	10.3	9.3	3.4	4.8
SNc	39.9	x	76.7	1.3	7.9
SNr	39.4	83.5	x	9.8	13.6
GPe	7.8	0.8	5.3	x	73.7
GPi	12.9	5.5	8.7	85.6	x
Total	100	100	100	100	100

*Each column shows the percentage of tracks connecting two nuclei, relative to the total number of direct connections through this nucleus*.

#### STN

**Figure 3:**
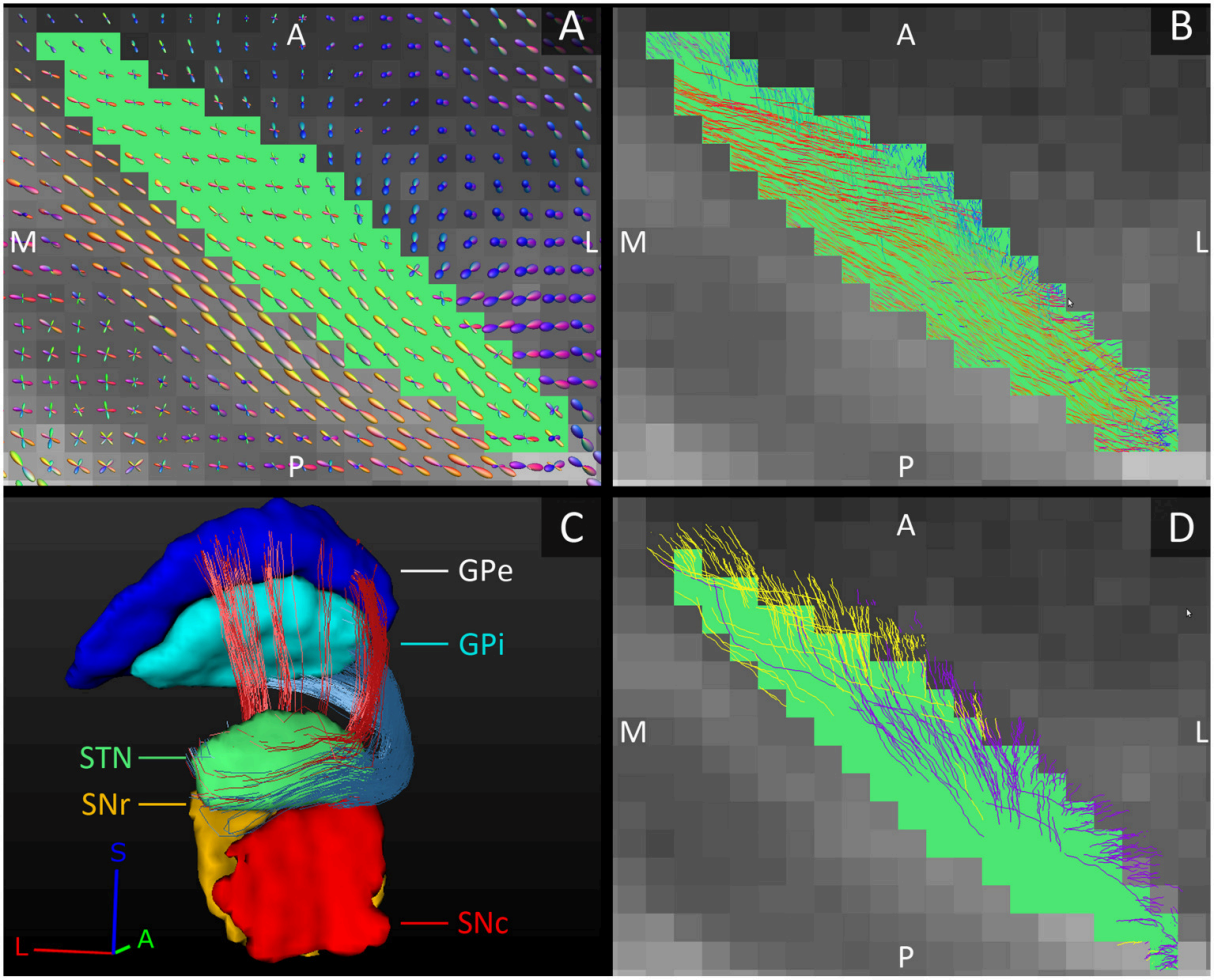
**Orientation of the fiber tracks of the subthalamic nucleus. (A)** The diffusion direction within the STN (green, axial plane) and its surrounding structures. **(B)** The fibers tracked within the STN. In **(A,B)**, the FOD-glyphs and tracks are color coded for orientation. **(C)** Reconstructed tracks directly connecting the STN to the GPe (red tracks) and to the GPi (blue tracks). Note that it is difficult to appreciate the actual number of tracks in the figure. **(D)** The STN (green, axial plane) and the fiber tracks connecting it to the SNc (purple tracks) and to the SNr (yellow tracks). A, anterior; L, lateral; M, medial; P, posterior; S, superior.

The fiber tracks within the STN ran from its posterolateral to its anteromedial side. This is in line with the direction of the dendrites that also arborize along this main axis (Sato et al., 2000). Toward the anteromedial side of the STN, the tracks fanned out in two directions, slightly more medial and slightly more lateral than their initial direction. This fiber direction within the STN also clearly separated it from the laterally bordering internal capsule whose fibers run inferior-superiorly (see Figures 3A,B).

#### STN-GP connections

The GPi is classically considered one of the output structures of the basal ganglia, receiving excitatory input from the STN (Nambu et al., 2000). These connecting fibers were successfully reconstructed in this study and were found to leave the STN at the anterior and medial side, circle anteriorly around the internal capsule and enter the GPi at its anterior and inferior borders, putatively following the course of the ansa lenticularis (Figure 3C). The STN has also been shown to excite the GPe (Nambu et al., 2000). These connections to the GPe were also reproduced and coursed either anteriorly around the internal capsule, in a bundle superior to the fibers connecting to the GPi, or they coursed straight through the internal capsule to the GPe's medial border (Figure 3C).

#### STN-SN connections

Just like the GPi, the SNr is classically considered one of the output structures of the basal ganglia receiving excitatory input from the STN (Nambu et al., 2002). In addition to these known connections between the STN and SNr, in this study we also found connections between the STN and SNc. The number of tracks found to directly connect the STN with the SNr and with the SNc was approximately the same. The STN borders the superior boundaries of both the SNc and the SNr. Not surprisingly, the tracks left the STN at its inferior lateral border and entered the SN at the superior border, where these two structures meet. The tracks to the SNc left the STN more anteriorly than the tracks to the SNr (see Figure 3D).

**Figure 4:**
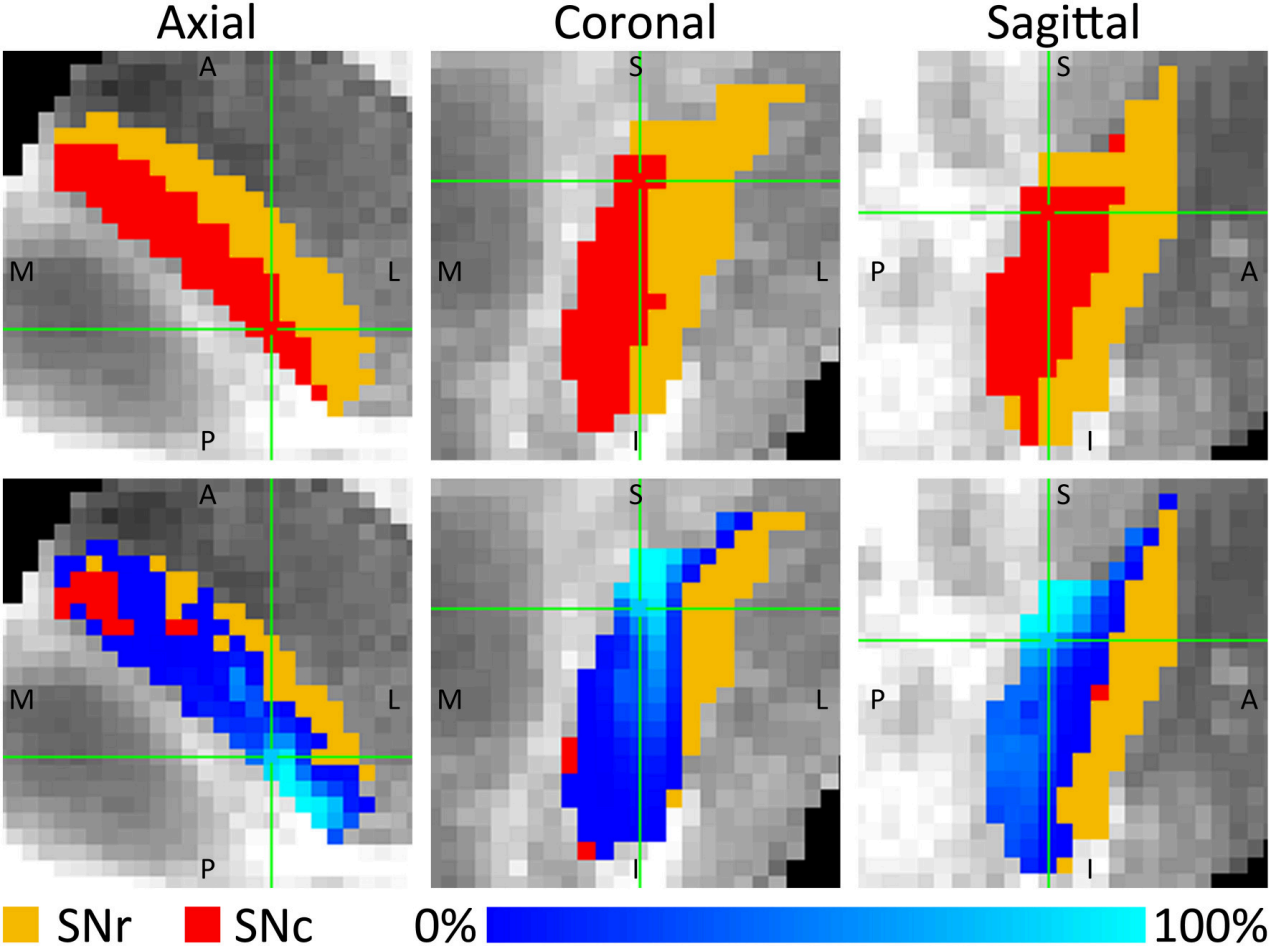
**Percentage of fiber tracks from the STN that entered the SNc (red) and SNr (orange)**. The **(Top)** row shows three slices through the SNc and SNr. The **(Bottom)** row shows these same slices, with on top of it, for each voxel, the number of tracks connecting that voxel with the STN expressed as a percentage of the total number of tracks through that voxel. A, anterior; I, inferior; L, lateral; M, medial; P, posterior; S, superior.

The tracks entered the SNc throughout its entire superior border, whereas the connections to the SNr mainly connected to the posterior part of its superior border. For both parts of the SN, the tracks were concentrated in the posterior parts of the SNc and SNr, but in the SNc a small proportion of the tracks also fanned out throughout a large part of the SNc (see Figure 4). This means that although the percentage of STN tracks that reached the SNc and SNr (either directly or indirectly) are similar (30.2 and 28.6%), within the SNc they connected to a larger volume than within the SNr.

#### SNc-SNr connections

**Figure 5:**
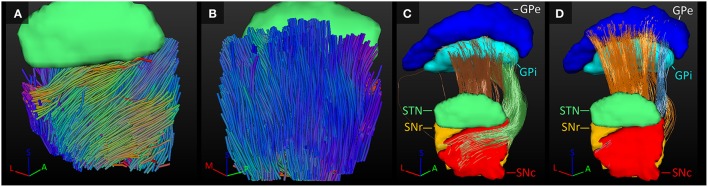
**Fiber tracks of the SNc and SNr. (A,B)** Fiber direction within the SNc **(A)** and SNr **(B)** color coded for orientation. **(C)** Fibers tracked between the GPi and SNr (brown tracks) and SNc (green tracks). **(D)** Fibers tracked between the GPe and SNr (orange tracks) and SNc (blue tracks). A, anterior; L, lateral; M, medial; P, posterior; S, superior.

Being two adjacent structures, many fiber tracks were found to connect the SNc with the SNr. Although in both segments the fibers traveled along the plane of the border of the both structures, interestingly, in the SNc the fibers traveled on average in superior-posterior direction, whereas in the SNr they traveled more diagonally from superior-medial to inferior-lateral (see Figures 5A,B).

#### SN-GP connections

The SNr and GPi represent a common structure separated by the internal capsule, both providing the output of the basal ganglia network. The SNr and GPi both receive, among other sources, inhibitory input from the GPe (Nambu et al., 2002; DeLong and Wichmann, 2007). This relation between the GP and the SNr was also found here, with connections tracked between both structures. Additionally we also found connections between the SNc and both elements of the GP.

Similar to the STN-GPi connection, the tracks from the SNc to the GPi circled anteriorly around the internal capsule to enter the GPi at the anterior part of its medial border. Some connections between the SNr and GPi followed this path as well, but others traversed straight through the internal capsule, entering the GPi more laterally along its medial border. The fiber tracks circling anteriorly of the internal capsule, left the SNr and SNc at their anterior side, but the fibers crossing through the internal capsule left the SNc at its lateral border (Figure 5C).

The connections between the SN and the GPe mainly traversed straight through the internal capsule. Relatively fewer fiber tracks connected the GPe to the SNc than to the SNr (1.3 vs. 9.8%) leaving only the anterior superior apex of the SNc connected to the anterior inferior border of the GPe. The fiber tracks of the SNr emerged from the anterior half of the superior border of the SNr and entered the GPe at its border with the internal capsule (Figure 5D).

#### GPe-GPi connections

**Figure 6:**
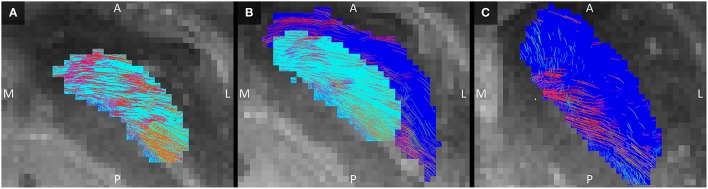
**Tracked fibers through the GPi (light blue) and GPe (dark blue) in three different axial planes**. The tracks are color coded for orientation and the planes are ordered from inferior **(A)** to superior **(C)**, with **(B)** inbetween. A, anterior; L, lateral; M, medial; P, posterior; S, superior.

The GPe inhibits the GPi (Nambu et al., 2002; DeLong and Wichmann, 2007). Connections between these two structures were indeed established here. In the GPi, the tracked fibers were mainly oriented from its anterior and medial side to its posterior and lateral borders (Figure 6A). Similarly, connections between the GPi and GPe started in the medial inferior part of the GPi and coursed in a slight curve along the GPe's longest axis toward the superior lateral part of the GPe (Figure 6B). Being a bigger structure, the fiber direction in the GPe was less homogeneous, with the majority of the fibers following the same direction as in the GPi, but part of the fibers left the GPe at its posterior border (Figure 6C).

### Connections to other structures and fiber tracks

Additional to these direct connections within the BG structures, direct and indirect connections with other basal ganglia structures and fiber paths were found as well (Table 2). From the GP, fiber tracks were reconstructed to the red nucleus and ventral pallidum although the percentages of reconstructed paths were low (< 3%).

**Figure 7:**
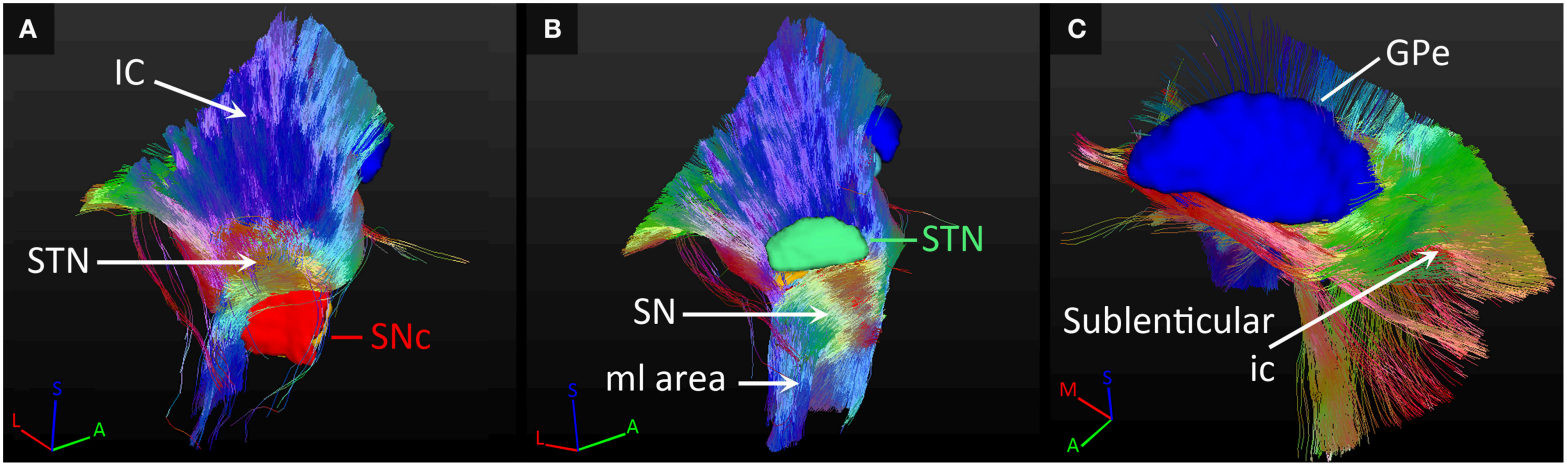
**Connections with fiber paths. (A)** Fiber tracks connecting the subthalamic nucleus (STN) and the internal capsule (ic). **(B)** Fiber tracks coursing inferiorly from the substantia nigra (SN). **(C)** Tracks fanning out along the course of the sublenticular internal capsule. All tracks are color coded for orientation. A, anterior; GPe, external globus pallidus; I, inferior; M, medial; ml, medial lemniscus; L, lateral; S, superior; SNc, SN pars compacta.

The most dominant of the connected white matter tracks were the strong connections to the internal capsule, which separates the STN and SN from the GP. In case of the STN for example, 95% of its tracked fibers either followed the internal capsule in superior direction, or crossed straight through it to connect to the GP (as explained in Section SN-GP Connections; see Figure 7A).

Also, fibers were tracked from these nuclei coursing inferiorly toward the brain stem. The SNc and SNr received more than 50% of their tracks from a fiber bundle located inferiorly in the area of the medial lemniscus. Many of these tracks continued to follow the course of the internal capsule in superior direction (see Figure 7B).

Fiber tracks that left the GP at its lateral side, continued to fan out along the path of the sublenticular internal capsule, thereby also indirectly connecting the SN and STN to this fiber path (see Figure 7C). The SN was also connected to a fiber bundle that coursed laterally upwards around the internal capsule to follow the course of the sublenticular internal capsule. Finally, a small amount of fiber tracks was found connecting the GP to the bordering anterior commissure, to the areas medial of the STN and the red nucleus, and to the putative interthalamic adhesion.

## Discussion

We performed high resolution tractography of a formalin-fixed post mortem human brain sample including the substantia nigra, subthalamic nucleus, and globus pallidus. With a diffusion weighted resolution of 0.5 × 0.5 × 0.5 mm^3^ isotropic, we generated detailed reconstructions of the connections of these nuclei.

Diffusion weighted imaging depends on the passive diffusion of water molecules, also called Brownian motion, that also occurs in post mortem tissue. An important advantage of *ex vivo* diffusion weighted imaging over *in vivo* imaging is the possibility of long scan times. This again facilitates diffusion images with high resolution, high contrast, and low noise. Compared to a resolution of 2 × 2 × 2 mm^3^ isotropic, which is common practice for *in vivo* diffusion imaging, we were able to investigate the brain's connections with a 64 times higher resolution, allowing for investigation of the connections of basal ganglia in great detail and specific to humans.

A few technical considerations have to be taken into account. Firstly, tractography, and especially probabilistic tractography, is sensitive to false positives (reconstructed tracks do not match actual fiber paths) and false negatives (actual fiber paths are not reconstructed). Therefore, one should be cautious when interpreting these results, and in particular any asymmetry in the connectivity values between structures, especially when a ground truth is missing. Also, compared to previously reported volumes of the left STN (Lambert et al., 2012) and GP and SN (Ahsan et al., 2007) measured with *in vivo* MRI, our measured volumes are on average 66.7% smaller, indicating shrinkage of the specimen potentially related to the formalin fixation (Quester and Schröder, 1997; Schulz et al., 2011). However, this shrinkage is not expected to affect the relative structural organization of the tissue. Indeed, the sample analyzed here included a few well described fiber bundles, including the anterior commissure and the internal capsule. The reconstructed fibers tracked through them corresponded well with known anatomy, which supports the validity of the remaining tracked connections that are not commonly established. Furthermore, it is likely that short distance connections are overrepresented because they are easier to track than long distance connections. The observed STN-GPi connections (17.2% of all tracks through the STN), for example, are fewer than the STN-SNr connections (28.6%). This could reflect a relatively important role for the STN-SNr connection in humans, or it could be an overestimation due to its shorter distance. The same holds for the many projections found between the GPi and GPe. Compensation methods have been proposed (Morris et al., 2008; Brunenberg et al., 2012), but were not used in this study to prevent the risk of overcompensation resulting in too many long-distance connections and too few short distance connections. Additionally, the direction of the connections cannot be inferred with tractography techniques. This directionality is already known for many of the basal ganglia connections; however, it is impossible to verify this with diffusion weighted tractography. It is therefore important to realize that the used descriptions of the courses of the tracks do not reflect the directionality of the projections. For example, the mentioned GPe-STN connections, may also reflect known STN-GPe connections (Kita et al., 2005). Similarly, it cannot be inferred whether the connections are inhibitory or excitatory. Finally, the STN, SN and GP all had many tracks following the internal capsule in superior direction. These tracks could be carrying the connections to the striatum or cortex as incorporated in the basal ganglia model (Nambu et al., 2002; DeLong and Wichmann, 2007). However, the cortex was not included in this study, but it would be interesting to investigate them in a larger post mortem specimen.

We could reconstruct some of the commonly established projections and white matter tracks of the SN, STN, and GP such as the internal capsule, anterior commissure, and ansa lenticularis. Also, we could confirm, at a fine scale and specific to humans, the connections between the STN, SNr, GPi, and GPe, that have been previously established on the basis of rodent, non-human primates (Sato et al., 2000; Haynes and Haber, 2013), and a few human studies (Aravamuthan et al., 2007; Lenglet et al., 2012; Calabrese et al., 2015). Post mortem diffusion tractography studies in humans have previously been performed at ultra-high field, resulting in a high resolution map of the brain stem (Aggarwal et al., 2013) and a detailed reconstruction of the dentorubrothalamic tract (Calabrese et al., 2015). A tractography study about the connections within the basal ganglia was previously performed *in vivo* by Lenglet et al. (2012). The STN-GPe, STN-GPi, STN-SN, and SN-GPi that we find are in line with their results. However, some of the observed fiber tracks, such as the connections of the GP with the anterior commissure, challenge the existing concepts of the basal ganglia network. Although it is not our intention to change the current way of thinking, we would like to stimulate a discussion about some of the novel findings without being too speculative.

To reduce scan time and increase imaging resolution, only a small brain sample was scanned. Because the striatum was not completely incorporated in the specimen, it was disregarded in the analysis. However, the observed connections from the SNc within the internal capsule may represent some of the known connections to the striatum (DeLong and Wichmann, 2007; Obeso et al., 2014) as also observed in other anatomical studies (Mai et al., 1986). Less well described connections that we found are connections of the SNc with the STN, SNr, and GP. It should be noted however, that diffusion based tractography cannot infer if a fiber projects onto a structure or passes through it. It might therefore be that some of these reconstructed tracks from the SNc merely traverse through the STN or GP without projecting onto them. Indeed, in a non-human primate tracing study, Sato et al. noticed that projections from the STN to the SNr coursed through the SNc without projecting onto it (Sato et al., 2000). However, despite its under appreciation in the functional basal ganglia models, the observed SNc-STN and SNc-GP connections have been previously indirectly demonstrated with tyrosine hydroxylase (TH) stainings in human tissue (Cossette et al., 1999; François et al., 2000). In line with our study, Cossette et al. showed dopaminergic connections from the SNc that followed the ansa lenticularis to reach the GP at its ventral surface (Cossette et al., 1999). However, we did not observe the additionally demonstrated connections from the SNc following the lenticular fasciculus (Cossette et al., 1999). Regarding the fiber organization within the SN, a prominent fiber bundle (bundle K) has been described to run along the caudal border of the SNr, parallel to the cerebral peduncle to bend at the base of the SN (Hassler, 1937). Although this is in line with the main fiber direction that we observed within the SNr, it does not explain the observed SNc-SNr connections. They might arise from unknown connections between the two parts of the SN. In *in vivo* human studies with 1.5 or 3 T MRI, it is often difficult to distinguish the SNc from the SNr (Lehéricy et al., 2014), which renders it difficult to infer their separate connectivity patterns. Sometimes, the identification of these two parts is even performed *post-hoc*, based on their expected projection sites (Menke et al., 2010; Lenglet et al., 2012), which might explain why this connection has not been previously observed with *in vivo* MRI. Alternatively these SNc-SNr connections might arise from falsely tracked pathways due to partial volume effects; voxels at the borders of a structure likely contain multiple types of tissue or fiber bundles from two distinct but bordering structures. Two other remarkable connections that we observed were the connections of the GPi with the interthalamic adhesion and of the GPi and GPe with the anterior commissure. Both these structures bridge the left and right hemisphere, although the basal ganglia are generally assumed not to have contralateral projections. A few explanations might account for this. One is that the observed fiber tracks represent actual connections that are difficult to observe with histological techniques or that are absent in rodents. Another explanation is that they are the result of falsely tracked fibers. This is indeed supported by the low number of connected fibers and the encapsulation of the anterior commissure by the GP. Finally, the GP, STN, and SN all had connections coursing inferiorly into the brain stem. These fiber tracks were located near the known course of the medial lemniscus. However, the medial lemniscus has been documented to be separate from the basal ganglia, which makes it unlikely for the reconstructed fiber tracks to represent the medial lemniscus. It putatively represents an adjacent bundle connecting to the periphery. However, in the brain stem the fiber paths become denser, hindering both accurate reconstruction as well as accurate identification of the tracks.

## Conclusion

Ultra-high field post mortem MR imaging allowed for reconstructing the connections of the human subthalamic nucleus, substantia nigra, and globus pallidus in great detail. In addition to the previously established basal ganglia projections, novel connections were seen as well. The latter may contribute to a more detailed understanding of basal ganglia function.

## Author contributions

The data was acquired by BP, AR, VK, KU, AH, and YT, analyzed by BP, AR, KU, and MM, and interpreted by BP, AR, JM, MK, AH, AJ, BH, and YT. All authors were involved in drafting and/or revising the work.

## Funding

This study was supported by a grant of the Netherlands Organization for Health Research and Development (ZonMW, grant nr: ZonMW/116350003/JSTP) and European Research Council (ERC) grant 269853. AR was supported by an ERC Starting Grant (MULTICONNECT, #639938) and an NWO VIDI grant.

### Conflict of interest statement

The authors declare that the research was conducted in the absence of any commercial or financial relationships that could be construed as a potential conflict of interest.

## Supplementary material

Supplementary Video 1 **Direct connections between the STN (green), SNc (red), SNr (orange), GPe (dark blue), and GPi (light blue) in a 3D rotation visualization**. The tracks are colored as follows: STN-GPe, red; STN-GPi, blue; SNc-GPe, purple; SNc-GPi, green; SNr-GPi, brown; and SNr-GPe, orange.

## Abbreviations

EPI: echo planar imaging
FOD: fiber orientation distribution
GP: globus pallidus
GPe: external part of globus pallidus
GPi: internal part of globus pallidus
GRE: gradient recalled echo
PD: Parkinson's disease
SN: substantia nigra
SNc: substantia nigra pars compacta
SNr: substantia nigra pars reticulata
STN: subthalamic nucleus
TE: echo time
TR: repetition time.
